# Editorial for the Special Issue on Advances in Optoelectronic Devices

**DOI:** 10.3390/mi14030652

**Published:** 2023-03-14

**Authors:** Zichuan Yi, Hu Zhang, Mouhua Jiang, Jiashuai Wang

**Affiliations:** 1 School of Electronic Information, University of Electronic Science and Technology of China, Zhongshan Institute, Zhongshan 528402, China; 2 School of Electronic Science and Engineering (National Exemplary School of Microelectronics), University of Electronic Science and Technology of China, Chengdu 611731, China

Optoelectronic devices are fabricated based on an optoelectronic conversion effect, which is a developing research field of modern optoelectronic technology and microelectronics technology [1]. In the 21st century, the global optoelectronic device manufacturing industry has achieved rapid development, and the market of optoelectronic devices is growing year by year. Optoelectronic devices are widely used in various fields such as optical displays, organic solar cells, lasers, and waveguides. They are an important part of information technology [2,3]. In order to expand the application scenarios and improve the performance of optoelectronic devices, many scholars have conducted research in related fields. This issue includes 12 papers that address various challenges and opportunities in algorithms, materials, and structures of optoelectronic devices. For example, in the field of optical displays, the response time and luminance of electronic paper could be improved by optimizing algorithms [4]. In the field of solar cells and waveguides, the conversion efficiency of solar cells and waveguide transmission distance could be improved by designing new optoelectronic materials and device structures [5,6]. The latest research advances in this Special Issue are as follows.

Electronic paper is a new device for image display by reflection, which is an important branch of optoelectronic devices [7]. The most widely used electronic paper is electrophoretic display (EPD). At present, ionic liquid was used as a charge control agent for electrophoretic particle modification, and high ionization 1-butyl-1-methylpiperidinium bromide mono ionic liquid was grafted onto the CuPc surface; then, blue electrophoretic particles were successfully prepared [8]. The modified blue particles had a high Zeta potential and electrophoretic mobility. The preparation process was simple, and the production cost was low, which contributed to the realization of a rich color display of EPDs. Moreover, the optimization of algorithms could also be used to improve the performance of EPDs. He et al. designed a driving waveform based on the principle of direct current (DC) balance [9]. The luminance curves of a unified reference grayscale phase were studied, and its driving time was obtained; at the same time, the duration of an erase stage was redesigned according to an original grayscale. The results showed that the response time could be effectively shortened. In addition, a three-color EPD could be prepared by adding red particles to an EPD [10]. In order to solve the problem of red ghost images, Wang et al. analyzed the spatial location distribution of red particles in grayscale transformation [11]. The key factors of red ghost images generation were investigated, and a driving waveform was proposed based on the optimization of erase and activation stages. Residual red particles on the top of microcapsules were eliminated in a red erase stage, and a high frequency voltage was used to activate the particles. The red ghost images were effectively suppressed. Similarly, some scholars found that black and red particles could be separated by a damping oscillation voltage sequence. The red particles were purified, and the red saturation of the pixels increased [12]. However, EPDs have the defects of a low refresh rate, and it is difficult to realize the full color display. A new type of optoelectronic device, electrowetting display (EWD), has attracted the attention of related scholars. EWDs have the advantages of an extremely short response time and full color display [13]. In order to improve the performance of EWDs, pixel structures, driving algorithms, and display systems were optimized. Tian et al. established a three-dimensional EWD simulation model through Comsol Multiphysics simulation software and proposed a new multi-electrode pixel structure [14]. The structure was composed of four square sub-electrodes. First, oil was broken by applying a voltage to an outer sub-electrode. Then, the voltage was applied sequentially to its two adjacent sub-electrodes. Oil could be rapidly driven to a saturation contraction state. The simulation results showed that the response speed could be effectively improved by the proposed structure. For the driving algorithm of EWDs, a driving waveform based on a threshold voltage and an exponential function was proposed by Long et al. [15]. The threshold voltage was obtained by measuring the relationship between DC voltages and luminance. Oil splitting could be inhibited by the exponential function voltage, thus improving the aperture ratio. Furthermore, a separated reset waveform was proposed to inhibit oil backflow [16], and an instantaneous negative voltage could be achieved by adjusting a common electrode plate. So, trapped charges could be quickly released by the negative voltage. For the display system, a dynamic adaptive display system was proposed [17]. The driving model was dynamically adjusted according to the display contents. The luminance was increased for still images and the refresh rate was increased for dynamic images. The above research contributed to the realization of high-performance EWDs and promoted the development of optoelectronic devices in the field of optical displays.

In the field of lasers, the linewidth is one of key parameters for the performance of lasers [18]. Currently, the most commonly used measurement method is the delayed self-heterodyne/homodyne based on an unbalanced interferometer, which has the defects of a long time consumption and noise [19]. Zhao et al. designed a short fiber-based linewidth measurement scheme [20]. The effect of a noise floor on the linewidth was measured and analyzed, and the application of short delay fiber lengths in delayed self-heterodyne laser linewidth measurements was investigated. The experimental results showed that the accuracy of the linewidth measurements was effectively improved. Due to the advantages of an ultra-short pulse width and ultra-high peak power, lasers can be used to process silicon [21]. However, the lattice temperature variation characteristic of silicon was difficult to study. Yang et al. developed a simulation model of a picosecond laser heating silicon based on a two-temperature model equation and the Fokker–Planck equation [22]. The effect of picosecond lasers with different pulse widths on silicon was measured. It was proved that the silicon processing efficiency was significantly improved. In the field of solar cells, a low optoelectronic conversion efficiency was the main reason for their limited application. The optoelectronic conversion efficiency of perovskite solar cells (PSCs) increased from 3.8% to 23% within a decade, which demonstrated the great potential of PSCs [23]. In order to further improve the conversion efficiency, the effect of non-plasmonic and plasmonic metal particles on the photocurrent density and open-circuit voltage of PSCs was examined. The electron transport layers of plasmonic PSCs were prepared in different deposition parameters, and an efficient localized surface plasmon resonance-based plasmonic PSC was successfully developed [24]. The surface morphology and optoelectronic properties of PSCs were investigated by testing the light-harvesting efficiency and steady-state photoluminescence. The light scattering and charge separation of PSCs were improved. The experimental results demonstrated that the performance of solar cells could be improved by adding plasmonic particles to photo anodes.

Waveguide is a kind of medium device which can confine a light wave in or near its surface and guide the directional propagation of the light wave [6]. It is commonly used in optoelectronic integrated devices. In order to reduce transmission losses, Yin et al. designed and fabricated a polymer/silica hybrid waveguide thermo-optical variable optical attenuator (VOA), covering the O-band [25]. It was prepared by a simple and low-cost direct ultraviolet lithography process. The response speed and bandwidth were improved by applying multimode interferences in the Mach–Zehnder interferometer VOA. A power equalization function could be achieved by integrating VOA and coarse wavelength division multiplexing. In addition, the performance of the waveguides could be improved by designing new structures. Surface plasmon polaritons (SPPs) were introduced into the waveguide design by Wang et al. [26]. The strong coupling effect could be observed in silicon waveguide mode and metal SPP mode, and excellent waveguide characteristics could be achieved. Therefore, a hybrid waveguide based on metal SPPs was proposed. The waveguide consisted of two silver nanowires and a rectangular silicon waveguide, which achieved good waveguide characteristics with a long transmission distance and a small normalized effective mode area. Similarly, new structures could be used to enhance the performance of resistive memories and electro-optic (EO) modulators. The effect of the barium carbonate film thickness on the performance of resistive memories was studied; then, a parallel dual microdisks memory resonator was proposed [27]. A two-port network was constructed by the parallel alignment of resistance memory components and bus waveguides. The resistive memory is the most promising next-generation nonvolatile memory because it has the advantages of a low power consumption, high scalability, and high compatibility. As for an EO modulator, it is a device which uses EO crystals to modulate the phase, amplitude, intensity, and polarization state of optical signals [28]. Wang et al. designed a thin-film lithium niobate EO modulator based on photolithography-assisted chemo-mechanical etching (PLACE) technology [29]. The fiber-to-fiber insertion loss was reduced, and the bandwidth was increased. PLACE technology had the advantages of a competitive production rate and high fabrication uniformity, which expanded the application scenarios of optoelectronic integrated devices.

Optoelectronic devices are playing an indispensable role in production and daily life. The development of society has higher requirements for optoelectronic devices. This Special Issue presented the latest research methods and achievements of optoelectronic devices in the fields of optical displays, lasers, solar cells, and waveguides. The luminance, conversion rate, and waveguide transmission capability can be effectively improved by applying new materials and designing new driving algorithms and device structures. It provided the possibility for the realization of large-scale photonic integrated devices, such as artificial neural networks, optoelectronic integrated circuits, etc.

## Data Availability

Some data are available from the authors upon request.
